# Eugenol: A novel therapeutic agent for the inhibition of *Candida* species infection

**DOI:** 10.3389/fphar.2022.872127

**Published:** 2022-08-09

**Authors:** Mojtaba Didehdar, Zahra Chegini, Aref Shariati

**Affiliations:** ^1^ Department of Medical Parasitology and Mycology, Arak University of Medical Sciences, Arak, Iran; ^2^ Department of Microbiology, School of Medicine, Hamadan University of Medical Sciences, Hamadan, Iran; ^3^ Molecular and Medicine Research Center, Khomein University of Medical Sciences, Khomein, Iran; ^4^ Department of Medical Laboratory Sciences, Khomein University of Medical Sciences, Khomein, Iran

**Keywords:** eugenol, *Candida* species, new antifungal agent, biofilm, combination therapy

## Abstract

The high occurrence and mortality rates related to candidiasis emphasize the urgent need to introduce new therapeutic approaches to treat this infection. Eugenol, the main phenolic component of *Clove* and *Cinnamomum* essential oil, has been used to inhibit growth and different virulence factors of *Candida*, including strains with decreased susceptibility to antifungals, particularly fluconazole. The results showed that this compound could bind to *Candida* membrane and decrease ergosterol biosynthesis, consequently leading to cell wall and membrane damage. Additionally, eugenol not only reduced germ tube formation, which reduces nutrient absorption from host tissues, but it also increased the levels of lipid peroxidation and reactive oxygen species, which induces oxidative stress and causes high permeability in the fungal cell membrane. Eugenol inhibited *Candida* cells’ adhesion capacity; additionally, this compound inhibited the formation of biofilms and eliminated established *Candida* biofilms on a variety of surfaces. Furthermore, by disrupting fungal cell integrity, eugenol could boost the entry of the antifungal drugs into the *Candida* cell, improving treatment efficacy. Therefore, eugenol could be used in the clinical management of various presentations of candidiasis, especially mucocutaneous presentations such as oral and vulvovaginal infections. However, further investigations, including *in vivo* and animal studies, toxicology studies and clinical trials, as well as molecular analysis, are needed to improve formulations and develop novel antifungal agents based on eugenol.

## Introduction

Candidiasis is emerging as a significant challenge in the healthcare environment due to its high medical costs and high mortality rates (Amirrajab et al., 2016; Pristov and Ghannoum, 2019; Shariati et al., 2020). Recent studies reported systemic infections caused by *Candida* species (*Candida*) as the fourth leading cause of nosocomial bloodstream infections. Opportunistic, these fungi are responsible for 90% of all invasive infections (Spampinato and Leonardi, 2013; Marak and Dhanashree, 2018; Sakagami et al., 2019). Although this family contains at least 15 distinct species, five pathogens could lead to invasive infections with a high mortality rate in humans: *Candida albicans*, *Candida tropicalis*, *Candida parapsilosis*, *Candida glabrata*, and *Candida krusei* (Pappas et al., 2018).

*C. albicans* is the most frequent etiology of candidiasis in clinical settings that could lead to a range of harmless superficial mycosis to life-threatening invasive infections. Nevertheless, non-*C. albicans* species collectively could represent >50% of the bloodstream isolates in certain regions (Wisplinghoff et al., 2004; Kullberg and Arendrup, 2015). In this regard, *Candida auris*, a previously rare fungus, has been reported as a major pathogen in certain parts of the world (Chowdhary et al., 2017). Overall, *Candida* species reside in healthy hosts as commensal yeasts in different parts of the human body, such as the gut and skin. Moreover, they are detectable in up to 60% of healthy individuals. However, immunocompromised status could lead to these fungi invading the host tissues and disseminating to internal organs (Pristov and Ghannoum, 2019).

*Candida* pathogenicity depends on various virulence factors such as yeast-hyphal formation and secretion of proteolytic as well as lipolytic enzymes, along with biofilm formation. Among the mentioned virulence factors, biofilm community of *Candida* has a potential role in pathogenicity. This biofilm community has a three-dimensional structure comprised of hyphal and yeast cells within a self-secreted matrix of extracellular polymeric substances (EPS), consisting of proteins, polysaccharides, and nucleic acids (Abirami et al., 2020). Biofilms are easily formed on the surface of medical devices such as intravascular and urinary catheters, artificial valves, intrauterine devices, contact lenses, and any foreign objects or host surfaces. Accordingly, the biofilm community of *Candida* leads to persistent infections and reduced susceptibility to host immune responses as well as antifungal drug therapy (Lohse et al., 2018; Abirami et al., 2020). Furthermore, the surface-associated virulence factors, such as adhesins and degradative virulence enzymes, including phospholipases and proteases have distinguishable roles in attachment and invasion into the host cells, while also strengthening *C. albicans* pathogenicity by destroying the vital proteins of the skin and cell membrane lipids (Mayer et al., 2013).

The specific type and dose of antifungal medication used to treat invasive candidiasis usually depends on the patient’s age, location and severity of the infection, as well as immune status. Accordingly, various antifungals such as echinocandins (caspofungin, micafungin, or anidulafungin) and azoles (fluconazole) are recommended to manage and treat invasive candidiasis (Fisher et al., 2011; Arendrup and Patterson, 2017). However, azoles-resistance and cross-resistances to azoles and echinocandins in fungi are now considered one of the main challenges in treating fungal infections. In this regard, a higher prevalence of drug resistance in *Candida* has increased the candidiasis-associated mortality rate (Zore et al., 2011). Furthermore, other drawbacks such as high cost, side effects and toxicity, and lack of fungicidal efficacy limit the usage of antifungals (Kauffman and Carver, 1997). These limitations justify the development of new therapeutic approaches or the discovery of novel antifungals to inhibit *Candida*-associated infections. Bearing this in mind, plant-derived substances due to high efficacy, few adverse effects, and low cost have shown potential capacity to inhibit these fungi, even azole-resistant strains (Ahmad et al., 2010a; Abrahão et al., 2013; Nisar et al., 2021).

Eugenol or 2-methoxy-4-[2-propenyl] phenol, a phenolic aromatic compound mainly derived from *Cinnamomum* and *Clove* essential oil, is one of these natural compounds belonging to a novel class of microbiocidal phenylpropanoids and has been used for a long time as an analgesic in dentistry (Hassan et al., 2018; Wijesinghe et al., 2021). Eugenol can be synthesized by guaiacol allylation with allyl chloride or produced through a biotransformation process that involves microorganisms such as *Bacillus cereus*, *Corynebacterium* species, and *Escherichia coli* (Abrahão et al., 2013). Eugenol has also indicated anesthetic, neuroprotective, antidiabetic, insecticidal, analgesic, anti-inflammatory, and antifungal properties that make this compound a versatile natural ingredient that helps prevent and cure various disorders (Kozam, 1977; Reddy and Lokesh, 1994; Hassan et al., 2018; Nisar et al., 2021).

Eugenol leads to a better penetration of different drugs in the skin. This compound has also been used as a pesticide and fumigant to protect foods from microbial invasion such as *Lactobacillus* and *Listeria monocytogenes* during storage. Additionally, the United States Food and Drug Administration approved the use of eugenol and clove oil as a natural antiseptic and analgesic in dentistry and as a fragrance in soaps and cosmetics. Furthermore, this compound has been used as a flavoring substance in food and pharmaceutical products (Kamatou et al., 2012; Mohammadi Nejad et al., 2017).

Eugenol exhibits significant antifungal activity against various fungal species, including dermatophytes, *Aspergillus*, and *Candida*, which is primarily due to damage to the fungal cell envelope, biofilm community, and various virulence factors (Chami et al., 2005; Hassan et al., 2018). As a result, researchers are interested in using eugenol and synthetic analogues, resorting to various drug delivery systems to inhibit *Candida*, biofilm communities, and several cellular pathways associated with these fungi.

This review focuses on the interaction of eugenol and various plant species with a high eugenol content with different cellular pathways in *Candida* cells. Additionally, we discuss the synergistic antimicrobial activity of eugenol when combined with antifungal drugs to facilitate its widespread use in clinical practice. Notably, the present study undertook a Medline (*via* PubMed) search using the following search keywords obtained from the National Library of Medicine’s Medical Subject Heading (MeSH) terms, titles, or abstracts using Boolean Operators (and, or): “Eugenol” and “*Candida*” or “Candidiasis” or “*C. albicans*”.

## Inhibitory effect of eugenol against *Candida* species

### *In vitro* antifungal studies

Recent *in vitro* studies used eugenol to inhibit *Candida*. In this regard, *Marcos-Arias* et al. evaluated the antifungal effects of this substance on a collection of oral *Candida* isolates from denture-wearers. The results showed that eugenol inhibited *Candida* growth at the Minimum inhibitory concentration (MIC) range 0.03%–0.25%. Furthermore, this compound showed potential inhibitory effects at very low concentrations against the susceptible-dose-dependent *C. glabrata* and the fluconazole-resistant *C. krusei* isolates (Marcos-Arias et al., 2011).

Another investigation was also performed using *Clove* essential oil (EO), collected from *Syzygium aromaticum*, against a collection of *Candida* strains isolated from patients with recurrent oral candidiasis. Gas Chromatography/Mass Spectrometry (GC/MS) analysis indicated eugenol (85.3%) as the main component of the EO. This compound showed inhibitory effects against all the tested *Candida* strains (MIC 0.08–0.64 μl/ml), including fungi with decreased susceptibility to fluconazole. Notably, inhibition of ergosterol synthesis and flow cytometry analysis revealed that this EO could cause extensive lesion of the *Candida* cell membrane *via* a significant decrease in ergosterol quantity and germ tube formation (Pinto et al., 2009). Noteworthy, ergosterol is a specific fungal cell membrane component, and germ tube formation is generally considered one of the most important *C. albicans* pathogenicity mechanisms that enables the absorption of nutrients from host tissues by invading them (Pinto et al., 2008). Therefore, the interaction of eugenol with ergosterol and germ tube formation could inhibit *Candida*-associated infections.

Additionally, recent research has examined the interaction of eugenol with *C. albicans* (CA04) using various techniques. Electron microscopy showed multiple sites of action in *C. albicans,* such as injuries to cytoplasmic contents, cell membranes, and cell walls after treatment with 200 μl/ml of eugenol. Furthermore, *C. albicans* cells exposure to eugenol led to 50% and 76% of dead cells and reduction in ergosterol biosynthesis, respectively. Thus, eugenol’s ability to bind on the *C. albicans* membrane and decrease the ergosterol biosynthesis may be associated with the cell wall and cell membrane damage (Kerekes et al., 2013).

These data support the findings by Ahmad et al.(2010a) who explored the antifungal effects of eugenol and methyleugenol against various *Candida* isolates. The authors reported that these components blocked ergosterol synthesis at their MIC values. Methyleugenol, based on the concentrations required to suppress *Candida* growth, showed higher antifungal activity than eugenol (Ahmad et al., 2010b). Moreover, another study also indicated that eugenol changed the morphogenesis of *C. albicans* envelope (Braga et al., 2007). In this respect, the authors proposed that eugenol could be a promising anti-*Candida* agent because it interferes with the morphology of the envelope of *C. albicans* and prevents morphological transition to hyphal form and adhesion, consequently decreasing *C. albicans* ability to colonize host tissues and its pathogenesis (Braga et al., 2007).

Furthermore, Khan et al. (2011) indicated that eugenol and methyleugenol, in addition to the inhibition of ergosterol biosynthesis, induced oxidative stress and caused high permeability in the cell membrane. It is noteworthy to mention that, besides being essential to maintain functional integrity and structure of the membrane, ergosterol also inhibits lipid peroxidation (LPO). LPO is one of the most important expressions of oxidative stress induced by reactive oxygen species (ROS). In fact, ROS interact with unsaturated lipids and manufacture polar lipid hydroperoxides that may increase membrane fluidity by disorganizing hydrophobic phospholipids. Thus, eugenol and methyleugenol may increase the level of LPO and ROS, thereby inducing elevated levels of oxidative stress in *C. albicans* (Khan et al., 2011).

These results were in line with Gupta et al. (2018) findings, who reported that eugenol eliminated around 80% of *C. glabrata* clinical isolates biofilm from biomaterials and increased ROS generation, cell lysis, and ergosterol content in the plasma membrane. Nonetheless, this compound inhibited catalase, proteinase, and phospholipase activity in *C. glabrata* cells. Interestingly, different genes such as *FKS*, *KRE1*, and *AUS1* were downregulated in response to eugenol treatment, where these genes were associated with 1,3-β-glucan synthase, GPI-anchored protein, and sterol importer, respectively. Contrary to previous studies, eugenol increased ergosterol content in *C. glabrata* cells. The differences in the ergosterol content detected in this study compared to other studies could be attributed to the divergent structure of *C. glabrata* cell membrane in comparison to *C. albicans*. Furthermore, the difference in eugenol concentrations may lead to this differentiation; however, further studies are needed to clarify this (Gupta et al., 2018).

Finally, the findings of Ahmad et al. (2010c) study showed that eugenol significantly inhibited H + -ATPase activity and glucose-stimulated H + -extrusion in various clinical isolates of *Candida*. Notably, H + -ATPase inhibition causes intracellular acidification and cell death. Suppression of cell growth and H + -efflux by eugenol suggests that its antifungal characteristics are also due to its prohibiting activity on H + -ATPase. Therefore, the authors suggested that it would be useful to further study eugenol’s interaction with the purified PM-ATPase enzyme and evaluate its function in both pre-steady and steady-state (Ahmad et al., 2010c). To this end, other studies also used eugenol to inhibit *Candida* growth, as reported in Table 1. These studies also reported acceptable inhibitory effect for eugenol against *Candida* species.

**TABLE 1:** Other studies that have used eugenol to inhibit *Candida* species.

Year of publication (references)	Plant source	*Candida* species (other microorganisms)	Combination of eugenol with other antifungal agents	Outcome
1982 Boonchird and Flegel, (1982)	NR	*C. albicans Cryptococcus neoformans*	NR	Inhibition and retardation of growth and germ tube formation
2007 Chaieb et al. (2007)	*Eugenia caryophyllata*	Various *Candida* species	NR	Showed inhibitory effects
2008 Lopes-Lutz et al., 2008	*Artemisia dracunculus*	*C. albicans* (various microorganisms)	NR	Showed inhibitory effects
2008 Matasyoh et al. (2008)	*Ocimum gratissimum*	*C. albicans* (Gram-positive and Gram-negative bacteria)	NR	Showed inhibitory effects
2011 Fontenelle et al. (2011)	NR	C. albicans *C. tropicalis* (Microsporum canis)	NR	Indicated *in vitro* antifungal activity against Soft-enter *M. canis* and *Candida* species
2011 Zore et al. (2011)	NR	*C. albicans*	Showed excellent synergistic activity with fluconazole	Killed 99.9% inoculum within 7 min of exposure and inhibited germ tube induction
2012 Fabri et al. (2012)	*Mitracarpus frigidus*	*C. albicans* (*Staphylococcus aureus Bacillus cereus, Pseudomonas aeruginosa and Enterobacter cloacae*)	NR	Indicated a strong antifungal effect
2012 Cecchini et al. (2012)	*Achillea ligustica*	*C. albicans Bacillus cereus* and *Streptococcus pyogenes*	NR	Showed inhibitory effects
2013 Thosar et al. (2013)	NR	*C. albicans* (*Staphylococcus aureus, Enterococcus faecalis, Escherichia coli*)	NR	Showed inhibitory effects
2020 Yassin et al. (2020)	*Syzygium aromaticum*	*C. albicans, C. glabrata C. tropicalis*	NR	Showed inhibitory effects

**FIGURE 1:**
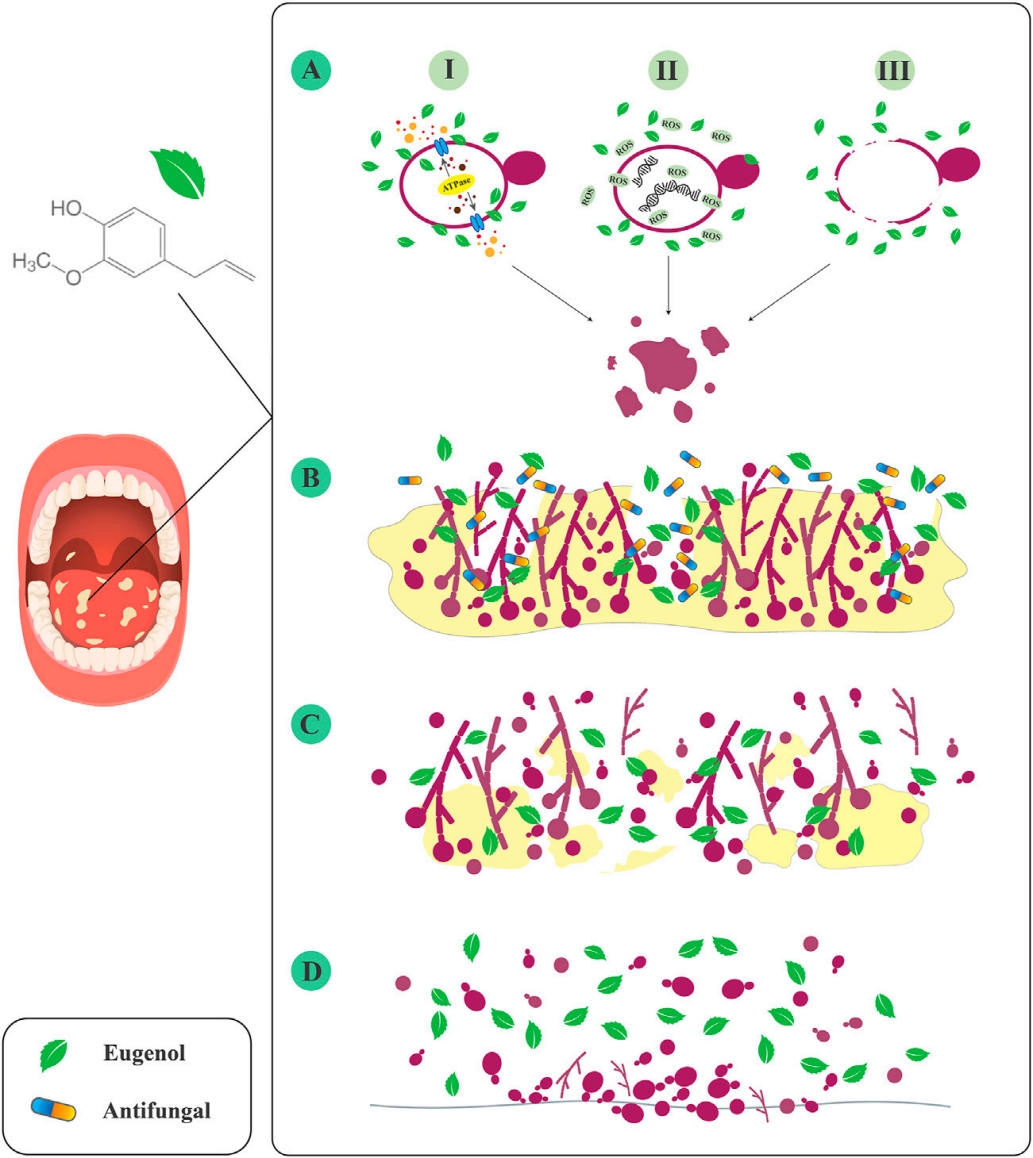
Inhibitory effects of eugenol against *Candida* species. **(A)** I: inhibition of plasma membrane ATPase. II: ROS production. III: disruption of cell structure. **(B)** Increase the penetration of antifungals to the deeper layers of biofilm. **(C)** Destruction of mature biofilm. **(D)** Inhibition of *Candida* attachment to the various surfaces.

Collectively, eugenol may inhibit *Candida* growth and pathogenesis *via* the following mechanisms: suppression of plasma membrane ATPase, which may play a role in hydrolytic enzyme secretion, ROS production, and apoptosis; disruption of *Candida* cell structure by binding to and removing membrane ergosterol; and disruption of gene function that may play a role in membrane biosynthesis (Figure 1) (Gupta et al., 2018). However, more *in vitro* and molecular studies are needed to understand the exact interaction of eugenol with various *Candida* cellular pathways.

### Anti-biofilm effects

The biofilm community of *Candida* is resistant to various antifungal treatments and environmental conditions. In this regard, *Candida’s* ability to form biofilm on abiotic and biotic surfaces is considered one of its most important virulence factors. Extracellular DNA and Exopolysaccharides (EPS) decrease antifungal penetration to the deeper layers of the biofilm, which is a serious concern that is aggravated by the emergence of azole-resistant isolates and the selection of *Candida* with decreased antifungal susceptibility (Williams et al., 2012; Cirasola et al., 2013). Since the biofilms’ resistance to common antifungal drugs has become more widespread in recent years, more investigations should be performed to produce novel, inexpensive, non-toxic, and effective treatment approaches by mainly controlling biofilm-associated infections. Accordingly, recent studies have focused on eugenol’s ability to inhibit *Candida* biofilm formation.

The results of He et al. (2007) study showed that eugenol could suppress the adhesion capacity and biofilm community of *C. albicans*. Additionally, the treatment of this fungus with this compound led to scant biofilms with inhibited filamentous growth. Notably, *C. albicans* is a dimorphic fungus that can switch from the yeast to filamentous phase. The filamentous phenotype of this fungus is essential for pathogenicity and could have a main role in producing the spatially organized architecture seen in mature, highly structured *C. albicans* biofilms (López-Ribot, 2005). Thus, these results proposed that eugenol could affect *C. albicans* cells’ morphogenesis and inhibit biofilm formation, thus attenuating this fungus invasive capacity (He et al., 2007).

Furthermore, molecular docking in another study showed the interaction of eugenol with Als3 (El-Baz et al., 2021). The Als adhesive proteins are one of the most extensively studied virulence characteristics of *C. albicans*, with the elimination of Als3 resulting in a considerable decrease in fungal adhesion capacity. The binding capacity of other natural compounds such as cinnamaldehyde was lower in comparison to that of eugenol (Hoyer and Cota, 2016). Thus, the interaction of eugenol and Als3 could be a promising finding for using this compound to inhibit *C. albicans* adhesion and biofilm formation (El-Baz et al., 2021).

In addition, eugenol*,* as a significant constituent of *Cinnamomum*, has indicated encouraging antibiofilm effects against *Candida*. In a study performed in 2018, the authors used *Cinnamomum zeylanicum* EO to inhibit *Candida* biofilm. The results showed that *C. zeylanicum* EO (500 μg/ml) remarkably suppressed the formation of *Candida* biofilm and inhibited monospecies (*C. tropicalis*) as well as multispecies biofilm community formed by these fungi. On the other hand, this EO at 1,000 μg/ml did not reduce human red blood cells viability. According to phytochemical evaluations, eugenol was found as the main component (68.96%) of the EO extracted from *C. zeylanicum* Blume leaves (Rangel et al., 2018). In another study, eugenol was also reported as the main compound (77.22%) of *Cinnamomum verum* EO. This compound suppressed initial adhesion, germ tube formation, and biofilm progression of *Candida*. Furthermore, microscopy evaluation demonstrated cell wall damages, diminished hyphal formation, and cellular shrinkages in *Candida* cells after treatment with this EO. On the other hand, no lethal effect of *C. verum* EO was observed using the *Galleria mellonella* experiment model at the various concentrations tested (Wijesinghe et al., 2020).

Wijesinghe et al. (2021) also reported eugenol as the main compound of *C. verum* EO. This EO destroyed the established biofilm community as well as hyphal production in *Candida*, and also led to cell wall damages and cellular shrinkages in these fungi. Nevertheless, *C. verum* EO did not show any effects against HaCaT (aneuploid immortal keratinocyte cell line from adult human skin) cells (Wijesinghe et al., 2021). Therefore, eugenol would destroy the cell wall integrity of *Candida* cell and suppress the biofilm community of this fungus. Furthermore, recent studies have reported a low cytotoxicity effect for this compound against different human cells.

Additionally, eugenol could also be used for the treatment of oral disorders that are associated with *Candida* biofilm. In this regard, in a study conducted by Jafri et al.(2019) this compound was used to suppress single and mixed biofilms of *C. albicans* [resistant to ketoconazole and itraconazole, as well as amphotericin B (AMB)] and *Streptococcus mutans.* The interaction of these two significant oral pathogens may result in recalcitrant and resistant infections in the oral cavity, thereby increasing the complexity of oral infection management. Microscopy evaluation indicated that eugenol caused cell shape alteration, less cell aggregation, as well as disarrangement of the single and mixed biofilms. Thus, at sub-MICs (100 μg/ml), eugenol significantly suppressed single and mixed biofilms formed by the drug-resistant strains of two oral pathogens (Jafri et al., 2019). Another investigation also reported inhibitory effects for eugenol against the planktonic and biofilm community of *C. tropicalis* and *C. dubliniensis* (dose-dependent and fluconazole-resistant strains) isolated from the oral cavity of HIV positive patients. No metabolic activity was detected in the biofilm after 24 h of treatment with eugenol (500 μg/ml); further, this compound markedly reduced biofilm cells on denture material surfaces. Also, eugenol significantly avoided the adhesion of *Candida* to the polystyrene and HEp-2 cells; thus, the authors proposed that eugenol may have an additional beneficial effect in the treatment of local candidiasis (de Paula et al., 2014).

Accordingly, the results reported in the mentioned studies showed that eugenol not only could potentially suppress the adhesion capacity of planktonic cells of *Candida,* but also could have a fungicidal activity against this fungus. Furthermore, this compound inhibited biofilm formation and destroyed the established biofilm of these fungi formed on various surfaces. Thus, eugenol is a natural compound with potential for non-toxic therapeutic application in the treatment of candidiasis by interfering with the most important virulence factors of *Candida* species, such as germ tube formation, adhesion to various host surfaces, and biofilm formation. However, the exact mechanism of antifungal and antibiofilm effects of eugenol against *Candida* was not clearly understood; therefore, further research is required.

## Combination therapy

Recent studies showed that new antifungal agents are needed to decrease the toxicity of conventional antifungals. Additionally, using combination therapy could improve the efficacy of various antifungals such as fluconazole (Pinto et al., 2009; Ahmad et al., 2010a). In this concept, the combined eugenol and different antifungal drugs were considered to inhibit *Candida* and their biofilm community. Indeed, a recently published study reported that eugenol, the main component of *Ocimum campechianum* Mill., could increase the efficacy of fluconazole against clinical *Candia* strains (Tacchini et al., 2021). Furthermore, Ahmad et al. (2010a) reported that eugenol and methyleugenol were active against fluconazole-resistant clinical *Candida* isolates. The interaction between methyleugenol and eugenol with fluconazole was synergistic in 92% and 90% of the fluconazole-susceptible strains, respectively. Additionally, 85% and 91% of the isolates resistant to fluconazole indicated synergistic effects of eugenol plus fluconazole and methyleugenol plus fluconazole, respectively. Notably, antagonistic interaction between the mentioned compounds was not detected in the strains tested (Ahmad et al., 2010a).

Moreover, previous studies considered the combination therapy of eugenol and fluconazole against *Candida* biofilms. An experiment conducted in 2014 reported a synergistic effect for combinations of fluconazole and eugenol against a planktonic community of *C. albicans*. The established biofilm of this fungus was highly resistant to fluconazole; while, sensitization of fungal cells by eugenol (sub-inhibitory concentrations) led to the prevention of biofilm formation at low fluconazole concentrations. The authors hypothesized that eugenol destabilizes the membrane and specific signal intervention. Hence, this compound caused sensitization of *C. albicans* biofilms, boosting fluconazole penetration and leading to the inhibition of biofilm formation (Doke et al., 2014).

Additionally, another study reported that pre-formed *C. albicans* biofilms showed ≥1,024× increased resistance to fluconazole, while biofilm formation did not cause tolerance to eugenol. This compound inhibited the biofilm formation of *C. albicans* and showed a synergistic interaction with fluconazole against biofilms formed by the test strains. The microscopy evaluation also showed eugenol interference with cell membrane integrity, as evidenced by shrinkage of the cell surface in biofilm cells. Therefore, the authors proposed that when eugenol has cidal activity against biofilm cells if combined with fluconazole, the drug’s fungistatic nature is converted to fungicidal (Khan and Ahmad, 2012).

In line with these observations, another investigation performed in 2020 in India reported that eugenol suppressed *S. mutans* and *C. albicans* mixed biofilms. The sessile MIC of fluconazole was increased up to 1000-fold over planktonic MIC. Notably, eugenol was highly synergistic with fluconazole against *C. albicans* single and mixed biofilms. The microscopy analysis also confirmed these findings and showed distorted cell structure, decreased matrix production, and elimination of single and mixed biofilm cells of *S. mutans* and *C. albicans* in treated samples with eugenol compared to untreated samples. Therefore, eugenol may disrupt cell membrane integrity and boost the drug entry into the microbial cell. This phenomenon increases the antimicrobial drug availability in the deeper layers and target sites of biofilm, consequently improving treatment efficacy (Jafri et al., 2020).

In addition to fluconazole, eugenol and other antifungals combined also showed promising results for inhibiting *C. albicans* growth. In this regard, Khan et al. (2012) reported that eugenol has a potential antifungal capability to inhibit fluconazole, itraconazole, and ketoconazole-resistant isolates of *C. albicans*. Furthermore, this compound showed remarkable synergy with fluconazole and AMB against the test isolates (Khan et al., 2012). The results of a recently published study also revealed that the combination of eugenol and sub-MIC (0.05 mg/ml) of AMB indicated many-fold higher anti-fungal effect against *C. albicans* compared to single component therapy. Scanning electron microscopy (SEM) showed the following characteristics for *C. albicans* cells after combination therapy: completely ruptured and shrank cells that aggregated as irregularly shaped material. Moreover, the authors reported that combination therapy induced ROS potentiation in *C. albicans* cells and with cellular damages, decreased mitochondria levels, and enhanced cytosol cytochrome C levels. Besides, the combination of eugenol and AMB resulted in an intense decline of intracellular Ca^2+‏^ concentration in *C. albicans* cells compared to the single treatments (Khan et al., 2019).

Noteworthy, the expansion of the inner mitochondrial membrane upon matrix swelling could damage the outer membrane, release the cytochrome C to the cytosol, and induce cell death (Wu et al., 2009). Therefore, these findings suggested that eugenol, in addition to *C. albicans* growth inhibition, could act synergistically with AMB (at less toxic doses) by interfering in the different cellular pathways of this fungus (Khan et al., 2019).

Another study reported a synergistic effect of eugenol in combination with voriconazole against voriconazole-resistant *C. tropicalis* and *C. krusei* isolated from the genital tract of mares (Sharifzadeh and Shokri, 2021). To this end, Dąbrowska et al. (2021) reported that eugenol indicated additive and synergistic activities with econazole and miconazole against clinical isolates of *C. albicans*, respectively.

Therefore, combining eugenol with various antifungal drugs could lead to several benefits such as decreased dose of drugs needed, boosted potency, and minimized toxicity, which ultimately helps suppress or eliminate biofilms and overcome fungal infections caused by drug-resistant *Candida* strains. However, the precise mechanism by which eugenol synergizes with various antifungals was not investigated in the studies mentioned above. Thus, additional molecular and *in vivo* studies are required to establish the practical utility of these combinations.

## Animal studies

Unfortunately, most *in vitro* observations of eugenol’s interaction with *Candida* cells have not been confirmed in animal models. However, some studies have evaluated this compound’s anti-*Candida* activity in *in vivo*, and this section discusses those studies.

Established murine models have been used for studying most important clinical forms of candidiasis such as vaginal, disseminated, cutaneous, and oropharyngeal. To this end, to evaluate *Candida* pathogenies and host responses to the treatment, mice treated with depleting antibodies and genetically modified mice (transgenics and knockouts) are generally used. Additionally, different strains of *Candida* including genetically modified laboratory strains and clinical isolates are used for analysis of *Candida* pathogenesis in animal models. *C. albicans* does not normally colonize the mice mucosa, though under different conditions of immunosuppression, disease can be induced (Solis and Filler, 2012; Conti et al., 2014).

Chami et al. (2004b) used eugenol (topical usage) to treat oral candidiasis induced by *C. albicans* in immunocompromised rats, where nystatin was used as a positive control treatment. Treatment with eugenol for eight consecutive days remarkably decreased the number of colony-forming units (CFU) sampled from the treated rats’ oral cavity compared to the control rats. Histopathologic examination (HE) revealed that the epithelium of the dorsal surface of the tongue of the untreated control rats was colonized with many fungal hyphae. Nevertheless, only a few focalized zones of the dorsal surface of the tongue were occupied by hyphae in eugenol-treated rats. Furthermore, no acute cytotoxicity effects were detected in eugenol-treated rats. Notably, HE revealed numerous hyphae in the tongue folds of rats treated with nystatin; however, this evaluation revealed no fungal hyphae in the fold following eugenol treatment. The authors suggested that this could be related to eugenol’s volatility characteristics, which allowed its penetration into unreachable zones, such as folds of the tongue (Chami et al., 2004b).

These data aligned with another investigation conducted in 2005 where the authors used eugenol to inhibit oral candidiasis in immunocompromised rats. The findings of this study also showed that eugenol had fungicidal effects against *C. albicans*. Moreover, treating rats’ oral candidiasis with this compound (eight consecutive days) led to a significant reduction of *C. albicans* colony count compared to the untreated control rats (Chami et al., 2005).

In another investigation, the authors also used eugenol to prevent and treat vaginal candidiasis caused by *C. albicans* in the immunosuppressed rat model. In this manner, to permit maximum adhesion of eugenol to the vaginal mucosa, a gelatinous suspension of 0.8% agar (as excipient) was used to treat rats through the intravaginal route. Ten days after infection, prophylactic treatment with this compound reduced *C. albicans* CFU in the vaginas of infected rats by 98.9%. Additionally, treatment with eugenol for seven consecutive days completely inhibited infection in 23% (2/9) of infected rats, whereas an 84% reduction in *C. albicans* colony count was detected in the vaginas of the other rats. HE of the lumina of the vagina also showed no *C. albicans* hyphae in all treated rats (Chami et al., 2004a). It should be noted that all of the above-mentioned candidiasis models in rats required an immunosuppressive host. Thus, the authors proposed eugenol as a potentially useful antifungal agent for preventing and treating candidiasis, particularly in patients with immunosuppressive conditions such as AIDS.

Finally, a recently published study evaluated the treatment effects of eugenol against fluconazole-resistant *C. albicans*-induced keratitis in the rabbit model. The results showed that 4 mg/ml of eugenol was the highest dose with non-toxic effects in the rabbit’s corneas. High-performance liquid chromatography (HPLC) analysis showed that the mentioned dose of eugenol is detectable in the corneal tissue; hence, this compound could penetrate through corneal epithelium that is an important obstacle for drugs penetration into the cornea. In addition, the clinical markers and HE showed significant lower levels of corneal neovascularization and clouding and conjunctival hyperemia in most rabbits treated with eugenol than the control group. Therefore, because most antifungal drugs are not applicable for the treatment of keratitis and have various side effects, the authors proposed eugenol as a safe and inexpensive antifungal agent that can be used topically for the treatment of keratitis caused by *C. albicans* (Hassan et al., 2018).

Therefore, as mentioned in the above studies, eugenol could be considered a potential antifungal agent with promising efficacy for treating different candidiasis presentations such as vaginitis, keratitis, and oral candidiasis. However, further molecular and toxicologic studies and clinical trials are required to determine the exact interaction of eugenol with eukaryote cells after prolonged exposure. Because non-albicans *Candida* species are becoming increasingly common, the antifungal effects of eugenol against these fungi should also be evaluated in future animal studies.

Studies regarding genotoxicity and cytotoxicity of eugenol are very controversial and limited. A dose of 2.5 mg/kg body weight of eugenol is regarded as safe by the Food and Agriculture Organization, while high concentrations of this compound could be harmful and pro-oxidative (Mohammadi Nejad et al., 2017; Ulanowska and Olas, 2021). For instance, 5–10 ml ingestion of clove oil led to acidosis and deep coma in a 2-year old boy (Hartnoll et al., 1993). Moreover, eugenol intravenous infusion (at 4 and 8 µl) caused acute respiratory distress with hemorrhagic pulmonary edema in rats (Wright et al., 1995). Also, eugenol could lead to allergies such as allergic contact dermatitis in some cases, particularly in dental workers (Mohammadi Nejad et al., 2017; Ulanowska and Olas, 2021). In this regard, research on eugenol and clove oil is still ongoing and extensive toxicity evaluations should be conducted to approve whether eugenol is safe for general public health.

## Drug delivery systems

Previous studies reported that eugenol is sensitive to degradation processes when exposed to oxygen, light, high temperatures, and humidity. Furthermore, this compound is highly volatile, slightly soluble in water, and unstable; as a result, the disadvantages listed above may reduce eugenol’s efficacy, biological activity, and stability (Karmakar et al., 2012; Shao et al., 2018). Therefore, considering the disadvantages of eugenol concerning solubility, instability, and volatilization, the use of different drug-delivery systems could be an alternative to solve these challenges and provide a stable, safe, and effective antifungal agent for clinical usage.

In this regard, Jacumazo et al. (2020) used nanocapsules and microcapsules containing eugenol coated with chitosan and carboxymethylcellulose to manage eugenol’s release enabling its use as an antifungal agent. This drug-delivery system indicated an inhibitory effect against *C. albicans,* and the MIC of eugenol was lower than those of the free agent. Thus, the authors suggested that nanocapsules with successive polysaccharide layers protect eugenol and improve its release (Jacumazo et al., 2020). Another investigation also reported that eugenol loaded with water-soluble βCD-grafted chitosan derivatives (QCD-g-CS) showed higher antimicrobial activities against *C. albicans* in comparison to the free compound. This enhanced antimicrobial activity could be attributed to the increased solubility of eugenol in the aqueous phase caused by the presence of QCD-g-CS, resulting in modified eugenol-microorganism interactions. Furthermore, both eugenol and chitosan have antimicrobial properties. Accordingly, the presence of both of them as inclusion complexes may lead to the synergistic effect on antimicrobial activity (Sajomsang et al., 2012).

Additionally, eugenol-loaded electrospun Polyacrylonitrile (PAN) nanofiber mats’ antifungal effects were evaluated against *C. albicans*. The findings demonstrated that eugenol release from nanofibers was gradual and continued slowly for 150 h. The PAN nanofiber lacked antifungal activity as a control sample, but these samples exhibited antifungal properties when loaded with eugenol. Hence, these data suggested that PAN nanofibrous mats containing eugenol could be considered an alternative therapeutic agent for localized drug delivery of eugenol due to its acceptable tensile properties, suitable drug release, uniform morphology, and antifungal properties (Semnani et al., 2018). Labib et al. (2015) also introduced another drug-delivery platform for treating local candidiasis in line with these results. The authors used Orabase, bioadhesive bases that have been used for pain relief in the handling of oral aphthous stomatitis, loaded with eugenol. This compound indicated significant antifungal function, acceptable physical features, slow-release pattern, and sensible mucoadhesion. Therefore, the incorporation of eugenol in Orabase, which has demonstrated efficacy in inhibiting local treatment of oral candidiasis, may aid in the design of future drug delivery systems (Labib and Aldawsari, 2015).

As mentioned in the above studies, diverse drug-delivery platforms with eugenol could provide novel agents to inhibit *Candida*-associated infection. Nonetheless, the precise mechanisms of these platforms interacting with candidal physiology remain unknown. Thus, additional research, including molecular evaluations, *in vivo* toxicity analysis, and clinical trials, are required before the widespread use of eugenol-drug delivery systems.

Finally, it is noteworthy that recent studies used eugenol-based derivatives to inhibit different cellular pathways in *Candida*. These studies have been reported in full in Table 2.

**TABLE 2:** Studies reporting synthetic analogues of eugenol to inhibit growth and *Candida* cells’ cellular pathways.

Year of publication (references)	Synthetic analogues of eugenol	*Candida* species	Mechanism of action
2012 Carrasco et al. (2012)	Eugenol derivative4-allyl-2-methoxy- 5-nitrophenol	*Candida* species	This compound did not inhibit the fungal cell wall synthesis or assembly
2015 Ahmad et al. (2015)	Eugenol-tosylate and its congeners (E1-E6)	Fluconazole resistant and susceptible *C. albicans*	The test compounds damage *C. albicans* ergosterol biosynthesis by reducing the gene-*ERG1* expression (one of the critical ergosterol biosynthesis pathways)
2015 Abrão et al. (2015)	Morpholine-based Mannich base of eugenol and the esters thereof	*Candida* species	The synthesized compounds indicated antifungal activity since most were more potent than fluconazole
2016 de Souza et al. (2016)	Eugenol Glucoside-based Derivative	*C. glabrata*	The test compounds suppressed *C. glabrata* growth better than fluconazole. Additionally, these compounds showed low cytotoxic activity against peripheral blood mononuclear cells
2018 Hipólito et al. (2018)	Eugenol-based glucosides	*C. tropicalis*, *C. krusei*	The test compounds were efficaciously involved in their high affinity with the active site of squalene epoxidase
2020 Lone et al. (2020b)	Eugenol tosylate congeners (ETC-1–ETC-7)	Fluconazole resistant and susceptible *C. albicans*	These compounds lead to the necrosis and apoptosis in *C. albicans* cells *via* the metacaspase-dependent pathway
2020 Lone and Ahmad, (2020)	Eugenol tosylate congeners (ETC-5, ETC-6, and ETC-7)	*C. albicans*	ETCs remarkably inhibited adherence, biofilm formation, proteinase, and phospholipase activity in *C. albicans* cells. Besides, tested compounds down-regulated the expression of various genes involved in the *Candida* cellular pathways
2020 Lone et al. (2020a)	Eugenol tosylate congeners (ETC-1 to ETC-7)	*C. albicans*	These ETCs target the biosynthesis pathway of the ergosterol in *C. albicans* via downregulation of gene-*ERG1* expression and impeding the lanosterol 14-α demethylase enzyme
2021 Magalhães et al. (2021)	Glucosyl-1,2,3- triazoles derived from eugenol and correlated phenols	*Candida* species (especially *C. krusei*)	The synthesized compounds suppressed CYP51, consequently destroying ergosterol synthesis in fungal cells

## Conclusion

Eugenol may be beneficial in the clinical management of candidiasis, particularly localized forms such as vulvovaginal and oral candidiasis, due to its fungicidal activity and inhibitory effect on germ tube formation. Due to the potent anti-biofilm capacity of eugenol against *Candida*, covering medical implant devices with this compound could be practical in preventing implant-associated *Candida* infections. The combined use of eugenol with various antifungals, especially fluconazole, could also be very helpful in combating infections caused by drug-resistant *Candida*. Nevertheless, although some studies have reported the molecular interactions of eugenol with the different *Candida* cellular pathways, further research is required to substantiate these findings. Additionally, more *in vitro*, animal models, and clinical trials should be designed for the exact evaluation of cell cytotoxicity due to long-term exposure to eugenol. Finally, it is proposed that various drug delivery platforms containing eugenol and other eugenol derivatives should be evaluated to develop effective and novel antifungal agents against *Candida* species.

## Abbreviations

AMB, amphotericin B; CFU, colony-forming units; EO, essential oil; EPS, Exopolysaccharides; GC/MS, Gas Chromatography/Mass Spectrometry; HE, Histopathologic examination; HPLC, High-performance liquid chromatography; LPO, lipid peroxidation; MIC, minimum inhibitory concentration; PAN, Polyacrylonitrile; ROS, reactive oxygen species; SEM, Scanning electron microscopy.
